# Utilization of healthcare services among Chinese migrants in Kenya: a qualitative study

**DOI:** 10.1186/s12913-019-4846-y

**Published:** 2019-12-26

**Authors:** Jialing Qiu, Duo Song, Juan Nie, Mengyi Su, Chun Hao, Jing Gu, Yuantao Hao, James N. Kiarie, Michael H. Chung

**Affiliations:** 1 Department of Medical Statistics & Sun Yat-sen Global Health Institute, School of Public Health & Institute of State Governance, Sun Yat-sen University, Guangzhou, China; 2 The International Committee of the Red Cross, Regional Delegation for East Asia, Beijing, China; 3 Department of Global Health, University of Washington, Seattle, USA; 4 Health Information Research Center & Guangdong Key Laboratory of Medicine, School of Public Health, Sun Yat-sen University, Guangzhou, China; 5 Department of Obstetrics and Gynecology, University of Nairobi, Nairobi, Kenya; 6 Department of Medicine, University of Washington, Seattle, USA; 7 Department of Epidemiology, University of Washington, Seattle, USA

**Keywords:** Migrants, Health services utilization, South-south migration, Qualitative

## Abstract

**Background:**

The number of Chinese migrants in Sub-Saharan Africa (SSA) is increasing, which is part of the south-south migration. The healthcare seeking challenges for Chinese migrants in Africa are different from local people and other global migrants. The aim of this study is to explore utilization of local health services and barriers to health services access among Chinese migrants in Kenya.

**Methods:**

Thirteen in-depth interviews (IDIs) and six focus group discussions (FGDs) were conducted among Chinese migrants (*n* = 32) and healthcare-related stakeholders (*n* = 3) in Nairobi and Kisumu, Kenya. Data was collected, transcribed, translated, and analyzed for themes.

**Results:**

Chinese migrants in Kenya preferred self-treatment by taking medicines from China. When ailments did not improve, they then sought care at clinics providing Traditional Chinese Medicine (TCM) or received treatment at Kenyan private healthcare facilities. Returning to China for care was also an option depending on the perceived severity of disease. The main supply-side barriers to local healthcare utilization by Chinese migrants were language and lack of health insurance. The main demand-side barriers included ignorance of available healthcare services and distrust of local medical care.

**Conclusions:**

Providing information on quality healthcare services in Kenya, which includes Chinese language translation assistance, may improve utilization of local healthcare facilities by Chinese migrants in the country.

## Background

With the strengthened diplomatic and economic ties between China and countries in Sub-Saharan Africa (SSA), more Chinese are migrating to the continent for economic reasons [1, 2]. They bring with them job opportunities, local transfer of knowledge and skills, and contribution to the local economy [3, 4]. The Chinese form a unique migrant group in SSA that differ from expatriates living in high-income countries or refugees in low- and lower-middle income countries (LMICs). To be specific, in the resource-limited African countries [5], Chinese migrants may face more challenges in access to health commodities (drugs and infrastructure) than migrants in high-income countries. However, on the other hand, Chinese migrants have relatively higher social economic status in the developing African countries, and this enables the Chinese more likely to afford the healthcare services of the private sectors compared to the local residents and refugees in LMICs. According to the World Bank in 2017, China is an upper-middle income economy while most SSA countries are categorized as LMICs [6]. At the same time, both China and SSA countries are geographically located in the global south and the Sino-African migration is part of the growing south-south migration, which is reported to account for the largest proportion of global movement in 2015 [7].

It is estimated that the number of Chinese migrants in SSA is between 580,000 and 1,000,000, but the exact figure is unknown [3, 4]. A 2012 survey by the China Africa Business Council of its 198 member companies in SSA estimated an average ratio of 1 Chinese worker to every 5 African workers [8]. Most of the Chinese in SSA are temporary labor migrants or contract employees of Chinese overseas companies. Others are independent entrepreneurs and merchants who undertake business ventures. One review of the literature described the economic and cultural impact of Chinese migrants in SSA [9], but none have explored their utilization of healthcare services and the barriers they may encounter in accessing care.

The objective of this study is: (1) to describe the types of healthcare services available to and used by Chinese migrants in Kenya; and (2) to investigate the barriers to accessing local healthcare services by Chinese migrants in the country.

## Methods

Between January 14 and March 18, 2016, we recruited participants in Nairobi and Kisumu, Kenya to participate in in-depth interviews (IDIs) and focus group discussions (FGDs). Nairobi is the capital and largest city located in the southern part of Kenya, and Kisumu is the third largest city located in the western part of Kenya. Compared to Nairobi, Kisumu has more rural sites with unfavorable quality of health services and also suffers higher prevalence of infectious diseases [10, 11]. Both cities have a lot of Chinese migrants living. The FGDs were carried out with Chinese migrants to identify the similarities and differences in their views of healthcare access in Kenya. This allowed us to gain an overview of the study phenomena and the norm among the target population. The information was complemented by the IDIs with Kenyan healthcare-related stakeholders and other Chinese migrants. In the IDIs, more in-depth information was obtained from these participants to explore complexity of the existing problems. In total, this study consisted of thirteen IDIs (with ten Chinese migrants and three local healthcare-related stakeholders) and six FGDs (with 22 Chinese migrants). The migrants in the FGDs did not participate in the IDIs simultaneously.

### Recruitment and participants

Both Chinese migrants and the Kenyan participants were recruited using purposive sampling. Purposive sampling is a technique widely used in qualitative research to identify and select information-rich individuals or groups for the most effective use of limited resources [12]. Chinese migrants were eligible if they were: (1) > 18 years old; (2) had Chinese nationality; and (3) worked and lived in Kenya for at least 6 months. The social acquaintances (e.g. staff in Chinese enterprises and Embassy in Kenya) of the Chinese investigators served as key informants and provided useful information on where to reach the potential Chinese participants. The investigators then visited different venues that Chinese migrants gather in Nairobi and Kisumu, such as the Chinese community around the China Embassy and Chinese state-owned enterprises. They identified potential study participants with different occupations and varying lengths of stay in Kenya. The migrants already participated were also asked to recommend someone they know who could provide rich information on the phenomena of interest, and the investigators visited them. Meanwhile, through the programs of University of Washington (UW) in Kenya, the UW team members facilitated the recruitment of local healthcare-related stakeholders in Kenya. A Kenyan doctor and a Ministry of Health (MOH) official and a pharmacist in the Chinese community were interviewed to determine their understanding of the challenges Chinese migrants faced. The potential participants were given a description of the study and study procedures. All the respondents were assured of confidentiality and anonymity. Then the respondents were asked whether they were willing to participate and whether they agreed to audio-taped for the study. Verbal consents were obtained from all the participants before data collection. Participants were recruited until data saturation was reached, which means to the point when there is enough data to ensure our research questions could be answered and no additional information to be provided [13].

### Interviews

Both the interview guides for the FGDs and IDIs were developed for this study and were made available in an additional file (see Additional file 1). The topics to guide the FGDs were semi-structured and adjusted iteratively based on the emerging themes of earlier sessions. Key topics including the general profile of Chinese participants, utilization of healthcare services, and healthcare service access barriers were discussed among the migrants. Individual participant information was collected including sociodemographic data, migration history, health insurance information, and medical history. Questions about utilization of healthcare services included whether the migrants sought healthcare, their experience of the healthcare services, and their preferred type of healthcare facility along with the reasons. All migrants were finally asked about barriers to local healthcare services. The topics for the Chinese migrants in the IDIs were similar to those used in the FGDs, but IDIs also included the questions for Kenyan participants. The Kenyan participants were asked about their experience of contacting any Chinese patients in their work and their views on the barriers to local healthcare service Chinese migrants might have.

All interviews with migrants were conducted in Chinese, while the interviews with Kenyan participants were conducted in English. The interviewers were two Chinese researchers who were experienced in qualitative interviews and were also familiar with the cultural background of the migrants. The IDIs were approximately 30 min to one hour in length. Similarly, the FGDs lasted for over a period of 30 to 60 min. All face to face interviews were conducted in conducive locations that were easily accessible to participants, such as the shops and offices. Three telephone interviews were conducted because the participants failed to keep the appointment or refused to meet with the interviewers. The interviews were recorded and transcribed, and notes were taken during the sessions. Transcripts were subsequently translated and independently checked by two separate study investigators.

### Data analysis

Data was coded using an inductive approach according to themes related to participants’ profiles, utilization of healthcare services, and healthcare service access barriers. An analytical framework was used to identify different dimensions of access barriers so that targeted interventions to each dimension can be designed [14]. Using this framework, themes of access barriers were divided into demand-side and supply-side barriers and then sorted into four dimensions: accessibility, availability, affordability, and acceptability. Accessibility described the extent the service was within reasonable proximity in terms of time and physical distance. Availability referred to whether a facility had services sufficient to meet the needs of patients. Affordability examined the formal and informal cost to obtain the healthcare and the ability of patients to pay for them. Finally, acceptability described how well the characteristics of the service matched the social and cultural expectations of the patient. Through an iterative process, a coding frame was developed and then refined as more data was accrued and analyzed. Accuracy of the codes was checked by separate investigators based on the transcripts. Areas of disagreement were discussed, and the consensus was reached by reviewing the transcripts during regular meetings.

## Results

### Participant profile

Ten Chinese migrants participated in IDIs and four of them were more than 50 years old. Of the ten participants, nine were male and one was female. There were five Chinese doctors, two Chinese state-owned company managers, two Chinese private company managers, and one Chinese embassy official. Among these Chinese participants, six had lived in Kenya for more than 20 years and three had utilized healthcare services in Kenya. Of the five Chinese doctors who were interviewed, one was a licensed Western Medicine doctor working in a Kenyan private hospital and four were Traditional Chinese Medical (TCM) doctors running their own clinics in Kenya. All the Chinese company managers and the Chinese embassy official had local health insurance or had their medical expenses covered by their employers. Three Kenyans were interviewed including one male Kenyan doctor at a private hospital, one female Kenyan pharmacist, and one male Kenyan MOH official (Table 1).

**Table 1:** Characteristics of in-depth interview participants

Ethnicity	Gender	Age range (years)	Employment	Position	Duration in Kenya	Medical reimbursement^a^
Chinese	Male	30–40	Private Company	Manager	7 years	Yes
Chinese	Male	30–40	State Company	Manager	3 years	Yes
Chinese	Male	40–50	Private Company	Manager	> 20 years	Yes
Chinese	Male	40–50	State Company	Manager	Unknown	Yes
Chinese	Male	40–50	Chinese Embassy	Official	Unknown	Yes
Chinese	Male	40–50	TCM Clinic	Doctor	> 20 years	Unknown
Chinese	Male	> 50	TCM Clinic	Doctor	> 20 years	Unknown
Chinese	Male	> 50	Private Hospital	Doctor	> 20 years	Unknown
Chinese	Male	> 50	TCM Clinic	Doctor	> 20 years	Unknown
Chinese	Female	> 50	TCM Clinic	Doctor	> 20 years	Unknown
Kenyan	Female	30–40	Pharmacy	Pharmacist	30–40 years	Unknown
Kenyan	Male	30–40	Kenyan MOH	Official	30–40 years	Unknown
Kenyan	Male	30–40	Private Hospital	Doctor	30–40 years	Unknown

^a^Having medical reimbursement here meant having local health insurance or having their medical expenses covered by their employers. The Chinese doctors and the Kenyans were not asked whether they had medical reimbursement

In the FGDs, all 22 participants were Chinese, between 25 and 50 years old, and comprised mainly of men (*n* = 15) (Table 2). Participants were interviewed in 6 FGDs. Three focus groups involved staff of Chinese state-owned companies (*n* = 13); one focus group included managers of a Chinese state-owned company (*n* = 3), and two focus groups were comprised of independent merchants (*n* = 6). The duration of living in Kenya among participants varied from six months to more than 20 years and seven of them had utilized healthcare services in Kenya. All state-owned company employees had local health insurance or had their medical expenses covered by employers, while only one merchant had local health insurance.

**Table 2:** Characteristics of focus group discussion participants

Focus Group	1	2	3	4	5	6
Total (*n*)	6	4	3	3	3	3
Age range (years)	25–40	25–40	25–40	30–40	30–50	25–40
Male (*n*)	3	4	2	3	2	1
Female (*n*)	3	0	1	0	1	2
Medical reimbursement^a^ (*n*)	6	4	3	3	0	1
Employment	State Co.	State Co.	State Co.	State Co.	Independent	Independent
Position	Staff	Staff	Staff	Manager	Merchant	Merchant

^a^Having medical reimbursement here meant having local health insurance or having their medical expenses covered by their employers

### Utilization of healthcare services

Most Chinese migrants in Kenya who fell ill treated themselves first, and then they sought health care at a TCM clinic or a Kenyan private facility. Visiting a Kenyan government hospital was not considered as an option but returning to China was considered if the disease was severe.

Almost all participants said that they self-treated by taking medicines from China. A few migrants explained that they felt perplexed about the usual dosage of the medicine that they bought in local stores and were concerned about the efficacy of the medicine: “*The dosage of the medicine is related to one’s body constitution and might be different between us and Africans. Using the medicine from China is more sensible (to get good efficacy)*.” [FG-3, Chinese State-owned Company Staff, Male]. Accordingly, some would subsequently seek care at a TCM clinic if ailments did not improve. TCM clinics were considered convenient and familiar and it was easy to communicate with a TCM doctor in Chinese: “*I would see a Chinese doctor when I get ill because it is easy to communicate. I know some of the Chinese doctors and Chinese clinics here*” [FG-6, Chinese Merchant, Male]. Some would attend the Kenyan private hospitals or clinics. Choosing care at Kenyan private hospitals or clinics was influenced by numerous factors including affordability, perceived quality, and distance. For example, private hospitals were considered if service was free or partly free due to health insurance or reimbursement by the company. In general, Chinese migrants preferred private hospitals and clinics to government facilities in Kenya because of perceived better quality of care, as one Kenyan doctor said, “*Yes, of course (I have received some Chinese patients), but not too many. They prefer private hospitals and sometimes they ask for better health service that means higher price as well*” [II-13, Kenyan Private Hospital Doctor, Male]. Most migrants did not trust the quality of government facilities and avoided them.

Returning to China was an important choice when encountering serious health problems. Many participants said that they would go back to China for treatment if the disease were severe since they were more confident of the quality of care in China. This included cases where surgery or hospitalization was needed.

“*The quality of medical service here is unfavorable. For example, if we need surgery, we think it is better to go back to China for care.”* [FG-1, Chinese State-owned Company Staff, Male].

### Healthcare service access barriers in Kenya

Demand and supply-side barriers were discussed under the themes of accessibility, availability, affordability, and acceptability (Table 3).

**Table 3:** Barriers to healthcare services among Chinese migrants in Kenya

Supply-side	Demand-side
Accessibility	
✓Traffic	
Availability	
✓Long waiting times	✓*Lack of knowledge of available services*
✓Lack of medications	
✓Quality of equipment	
Affordability	
✓High medical expenses	✓Unwillingness to buy health insurance
✓*Limited insurance packages for Chinese migrants*	
Acceptability	
✓*Language*	✓*Misconception and distrust of services*
	✓Perceived risk of infectious diseases

*Italic*: the main barriers to care

#### Accessibility

Chinese migrants generally did not cite geographic access to care as a problem. However, traffic was cited as a problem by one company manager when coming into the city from remote locations.

“*One problem for us is traffic. We live in a remote place and it takes us at least thirty minutes to drive to the designated healthcare facility*.” [FG-4, Chinese State-owned Company Manager, Male].

#### Availability

Long waiting times in clinics and hospitals due to staff shortage was emphasized. Some Chinese migrants indicated that essential medications were not always available and that medical equipment appeared out of date. However, the most severe problem is that many Chinese migrants were unaware of what healthcare resources were available in Kenya.

“*Sometimes the company staff would come to me for help on the phone and I would help them contact the doctor of a relevant medical department, such as neurosurgery or emergency. I think there really needs to be a service station that can offer counseling and referral suggestions for Chinese migrants.*” [II-8, Chinese Private Hospital Doctor, Male].

“*We don’t know where to buy medicine… We have limited knowledge of local hospitals or TCM clinics… I hope to know the information about healthcare facilities that provide qualified services and that the information includes the address, how to get there, and how to contact them.*” [FG-2, Chinese State-owned Company Staff, Male].

#### Affordability

The cost of healthcare services is generally much higher in Kenya than it is in China, where most rely on a national health insurance system that covers most of their needs. The Kenyan doctor and the pharmacist indicated that Chinese migrants seldom complained to them about the prices and were often affordable for the services. However, the migrants expressed that paying out-of-pocket for medical expenses in Kenya challenged their tolerance to a certain extent on what is affordable healthcare. Participants recounted the negative experience of paying what they considered to be unreasonably high prices for services including registration, examination, treatment, hospitalization, and the extra cost caused by misdiagnosis.

“*The service of the treatment is really expensive. My dental braces once fell off and it cost me more than 70 dollars for the care.*” [FG-2, Chinese State-owned Company Staff, Male].

*“[I have health insurance and I still believe that] the expense of [healthcare] services is definitely high. Last time I got a small surgery here for kidney stones. Later I was hospitalized for a long time because of severe infection and many other problems. I had to pay about two million Kenyan shillings, which is equivalent to 20,000 US dollars if I don’t have health insurance.”* [II-3, Chinese Private Company Manager, Male].

Since Chinese national health insurance does not cover medical expenses in Kenya, most Chinese migrants remain uninsured in the country. Many migrants refused to buy local health insurance because they were staying for only a short time and did not perceive that it was worth the cost. There did not appear to be health insurance packages in Kenya that targeted short-term Chinese migrants. Moreover, many understood that they could return to China for treatment that would be covered under the national health insurance plan if their condition were serious.

#### Acceptability

The language barrier was mentioned by nearly all participants who were interviewed in both the IDIs and the FGDs. Most Chinese were poor at English, let alone the medical terminology, leading to communication difficulties with healthcare workers in local hospitals and clinics. Friends or interpreters were often invited in person or on the phone to help with translation.

“*Chinese clients don’t speak English well... I hope they can speak better English or come with someone good at it, such as a translator. When I communicate with them, I try, struggle so hard to make myself understand.*” [II-11, Kenyan Pharmacist, Female].

“*I think the terminology of Western Medicine and their spoken English is kind of difficult to understand… Last time I went to the hospital to get a tetanus shot, I telephoned my friend and asked her to communicate with the doctor for me.*” [FG-6, Chinese Merchant, Female].

Another barrier in acceptability was misconception and distrust of the quality of healthcare services in the country. Chinese migrants were constantly comparing the quality of care in Kenya with that in China resulting in unreasonable suspicions fueled by rumors or the negative experience of others.

“*When our manager was ill last year, all of us did not believe the diagnosis in the Kenyan hospital and insisted on going back to China for care. But the doctor in China gave the same diagnosis. That’s the problem. We could not trust the diagnosis here because there was so much misdiagnosis we’ve heard.*” [FG-1, Chinese State-owned Company Staff, Female].

Some Chinese migrants utilized the local healthcare services in Kenya and thought it was good, but they preferred private facilities where the quality of care was perceived to be higher and the risk of infection to be lower. Some were concerned about the risk of acquiring infectious diseases such as HIV in Kenyan healthcare facilities.

## Discussion

To our knowledge, this is the first study to examine the healthcare services utilization of Chinese migrating to Kenya, under a setting of the south-south migration. In general, it was found that Chinese migrants preferred to treat themselves first and then sought care at TCM clinics or attended Kenyan private hospitals and clinics. Returning to China for care was an important option for many to address serious diseases. The main barriers to care were language difficulties, limited insurance packages, lack of awareness of what healthcare resources were available, and poor perception of the quality of local healthcare services.

Existing evidence shows that it is a common phenomenon for migrants to turn to health providers from their own country or to return to their home country [15], all of which can be understood as the migrants’ spontaneous strategies to get culturally and linguistically appropriate health services. Mexican migrants sought care from the Mexican health providers in the US not only for the physical problem but also to deal with the spiritual risk and gain emotional support [16]. The majority of the Latin American migrants in a qualitative study reported having utilized the healthcare services from the Latin American or Spanish-speaking doctors in London [17]. In this study, the participants visited the TCM clinics mainly because it is easy to contact and communicate with the doctors. As more TCM doctors in SSA establish their own clinics that provide an alternative treatment option for the migrants, it may be beneficial for both African and Chinese governments to work together to regulate the quality of TCM on the continent [18]. Ensuring the quality of healthcare services provided in TCM clinics would undoubtedly benefit the African community as well [19, 20]. Returning to China was also considered by many Chinese migrants and is consistent with the results of another study suggesting that this behavior was related to healthcare access barriers in the host country [15].

Kenya is a lower-middle income country [6], and its healthcare services are provided by the government sector, the private sector, and non-governmental organizations (NGOs) [21]. The government sector and NGOs serve most of the population including refugees while private facilities cater to paying patients or those with private health insurance [22, 23]. Overall, there remains a shortage of healthcare workers and health commodities in Kenya [21]. As the number of migrants in Kenya has rapidly increased, the Kenyan government has signed international agreements to extend healthcare to all population including the migrants [24]. In this study, barriers to accessing healthcare in Kenya by Chinese migrants are similar to barriers in more resource-rich settings such as lack of health insurance and language problems [25–27]. However, what may be highlighted in Kenya is the misconception and distrust of healthcare services, particularly in the government sector. Moreover, the relatively high prevalence of infectious diseases in SSA has increased the migrants’ fear of acquiring infectious diseases such as HIV in these local facilities [28]. It might be helpful to cope with these two demand-side barriers by increasing the migrants’ knowledge of quality health facilities in Kenya (this was also important to deal with their ignorance of available local healthcare services and we would describe more about it in later section) and strengthening their confidence in the healthcare services in Kenya through electronic and network dissemination of positive examples.

Problems understanding English was mentioned by most Chinese as the most challenging supply-side barrier to accessing healthcare in Kenya. This is similar to findings from other studies in the field and is regarded as an essential component in facilitating healthcare access among migrants [25, 29, 30]. Professional medical interpretation is widely advocated and several studies have reported its positive effects on improving health outcomes and reducing long-term costs [31]. However, providing translation services is a time-consuming and resource-intensive assistance and may not be feasible in the setting of constrained healthcare services in SSA [31]. Furthermore, improving communication requires not only language proficiency but also understanding of cultural and behavioral differences between Chinese patients and their Kenyan doctors [32]. In this sense, multifaceted efforts were needed to overcome the complexity of the language barrier and the interpretation services should also be evaluated whether they actually meet migrants’ expectations [29, 32, 33].

Finally, as the demand-side, many Chinese migrants were unaware of what healthcare services were available in Kenya and were unwilling to buy local health insurance to cover these costs. Information on available quality facilities and healthcare providers in Kenya needs to be provided to the migrants, guiding them to make sensible health decisions [14, 34]. These efforts could be supported by the numerous global health programs and healthcare training programs that China has implemented in SSA including Kenya [35]. Health information sharing can also be taken up by the Chinese Embassy in Kenya, Chinese community organizations in Kenya, and Chinese businesses that employ migrants. More private and community-based health insurance schemes are needed [36], and could be introduced to short- and long-term migrants to release their financial burden and protect them from the catastrophic expenditures.

### Limitations

There are several limitations to this study. First, this was a qualitative study that was limited in the number and types of participants, and therefore may be difficult to generalize to all Chinese migrants and their Kenyan healthcare providers. Second, the number of Kenyans who were interviewed was limited making it difficult to confirm their understanding of the healthcare challenges Chinese migrants might face. Third, we did not have a comparator Kenyan group to understand how many of the issues identified by Chinese migrants may have been challenges to healthcare access experienced by all Kenyans. Forth, there exist more important factors that influence the health access but are not fully explored in this study, such as the individual’s health literacy. Further research should consider how other factors influence the health access of Chinese migrants in Africa.

## Conclusions

In summary, migration from China, an upper-middle income country, to Kenya, an LMIC, raises unique challenges to the utilization of healthcare services. In many ways, these are similar to barriers faced in high-income countries including language difficulties, lack of health insurance, and ignorance of available services, and resulted in the same outcomes including reliance on self-treatment and attending TCM clinics. However, there are also challenges specific to this south-to-south relationship including distrust of public sector services due to limited resources and a heightened fear of infectious diseases given their relatively high prevalence. Further studies are needed to bridge these healthcare gaps as partnerships between China and SSA countries grow and Chinese migration to the continent increases.

## Supplementary information

**Additional file 1.** Interview guides.

## Data Availability

The datasets used and/or analyzed during the current study are available from the corresponding author on reasonable request.

## Abbreviations

FGD: Focus group discussion
IDI: In-depth interview
LMICs: Low- and lower-middle income countries
MOH: Ministry of Health
SSA: Sub-Saharan Africa
TCM: Traditional Chinese Medicine
UW: University of Washington
