# Supplementary material for: Utilization of healthcare services among Chinese migrants in Kenya: a qualitative study
Source: BMC Health Serv Res. 2019 Dec 26;19:995. doi: 10.1186/s12913-019-4846-y (PMC6933712; doi:10.1186/s12913-019-4846-y)
Supplement: Supplementary file 1 — Additional file 1. Interview guides. [file 12913_2019_4846_MOESM1_ESM.docx]

**Additional file 1: Interview guides for FGDs and IDIs.**

(This manuscript focused on participants’ responses of the questions in the *Health Services Utilization in Kenya* part)

**I. Interview Guide for Focus Group Discussions.**

This interview guide is divided into several main topics. Try to address each topic during the interview; however, you may end up changing the order depending on the low of the conversation. Under each topic, a number of sample probes are included. These are not intended as questions that must be asked during each interview. Rather, they meant to facilitate your probing. After each interview, please write down notes on additional main topics for further interviews, probes that provided good insight and any reflections on the interview content.

**Chinese Community in Kenya**

1. Based on your observations, how many Chinese migrants are working or living in Nairobi, Kenya? Where are they from in China? How about their ages? How about their nationality?

2. What are they working for?

3. Where is the Chinese migrants' living community in Nairobi, Kenya?

4. Are they usually living with their family members?

5. How are their feelings about Nairobi, Kenya? Will they plan to permanently resident here?

6. How do Chinese migrants social network here? Is there any social network place or platform here?

**The Health Status of Chinese Migrants**

7. Based on your observations, what are the common health problems among Chinese migrants here? (e.g. common diseases, vaccines, delivery, etc. )

8. Did any of your friends or acquaintance here have some health-related problems? What kinds of health-related problems? How do they deal with that? (e.g. injury, infectious diseases, fevers, etc.)

9. Did any of your friends or acquaintance here have some chronic disease problems? What kinds of chronic diseases? How do they deal with that? (e.g. hypertension, diabetes, etc.)

**The Health Services Utilization of Chinese Migrants**

10. What kind of health facilities do you prefer to go to usually? (e.g. Chinese Traditional Medicine Clinics, etc. )

11. Where do you get the medicine in Nairobi, Kenya?

12. Do Chinese migrants have health insurance for health services in Kenya? What kinds of health insurance?

13. How do Chinese migrants pay for health services or medicine in Kenya? Compared with in China, is the price higher or lower?

14. Do you know any resources or supports from China or Kenya are able to help your health utilization in Kenya? (e.g. clinics, services, insurance, payment, etc.)

15. What is the biggest barrier or difficulty for health seeking or utilization for Chinese migrants in Kenya?

16. What is the health utilization problem pressed for solution for Chinese migrants in Kenya?

**II. Interview Guide for In-depth Interviews.**

This interview guide is divided into several main topics. Try to address each topic during the interview; however, you may end up changing the order depending on the low of the conversation. Under each topic, a number of sample probes are included. These are not intended as questions that must be asked during each interview. Rather, they meant to facilitate your probing. After each interview, please write down notes on additional main topics for further interviews, probes that provided good insight and any reflections on the interview content.

**Topics for Chinese migrants:**

**Background information**

*Now I want to ask you a little bit about your background*

**1. Please tell me about a little bit about yourself**

*Probes:* (1) what brought you here? (2) what are your ethnicity (Han, Zang, Hui, etc.) and education level? (3) How long have you been in this city/country? How do you like this city/country? (4) Where do you live? (5) Who do you live with? Who else do you live with?

**2. Where are you from in China?**

*Probes:* (1) Which city? (2) Rural or urban? (3) What is your nationality?

**3. Do you have children?**

*Probes:* How many/ how old are they/ where do they live now/ what do they do/ are they married?

**4. What brought you to this city/country?**

*Probes:* invited by others (who) / needed a job/ there were no opportunities at hometown

**5. What kind of work have you done in the past?**

*Probes:* (1) Describe these jobs? What was your last job (2) How long did you work on these jobs? (3) The reasons that you come here?

**6. Do you plan to permanently resident here?**

*Probes:* (1) If yes, why? (2) If not, why?

**7. How about your social network here?**

*Probes:* (1) Do you meet your friends here in China or in Kenya? (2) How to meet new friends here? (3) Is there any social network platform for Chinese migrants in Kenya?

**Health-related problems**

*Now I want to ask you some questions about your health*

**8. Did you or your family members have some health-related problems here?**

*Probes:* (1) Who has the health problem? (2) What is the problem? (3) When did it happen? (4) How to deal with that?

**9. In general, how do you or your family members deal with health problems?**

*Probes:* (1) How to deal with acute diseases? fevers, injury, etc. (2) How to deal with chronic diseases? hypertension, diabetes, etc.

**The Health Services Utilization in Kenya**

**10. How do you or your family members self-treat here?**

*Probes:* (1) Where to get the medicine? (2) Where to buy medicine here?

**11.** **How do you or your family members seek health services here?**

*Probes:* (1) What kind of health facilities do you prefer to go to usually? (2) Why choose these facilities? (3) How far is it? (4) How's the quality of these health facilities? (5) Does it able to resolve your health problem? (6) How do you pay for the services there? (7) Do you have any health insurance, and will these facilities accept that?

**12. Do you or your family members know any resources or supports from China or Kenya are able to help your health utilization in Kenya?**

*Probes:* (1) Health insurance or reimbursement? (2) health policy or measures?

**13. What is the biggest barrier or difficulty for health seeking or utilization for you in Kenya?**

*Probes:* (1) Barriers or difficulties for self-treatment? (2) Barriers or difficulties for health services seeking?

**14. What is the health utilization problem pressed for solution for you or your family members?**

*Probes:* (1) For self-treatment (2) For health services seeking

**Topics for Kenyan healthcare-related stakeholders:**

**Background information**

**1. Please tell me a little bit about yourself**

*Probes:* (1) What is your job and position? (2) How long have you been in this city/country?

**2. Have you ever seen any Chinese clients during your work?**

*Probes:* (1) How many Chinese clients do you usually have every day? (2) What’s your impression on them? (3) How can you communicate with them?

**Chinese Community in Kenya**

**3. Based on your observations, how many Chinese migrants are working or living in Nairobi, Kenya?**

*Probes:* (1) How about their ages? (2) What brought them here? (3) What are the migrants’ ethnicity (Han, Zang, Hui, etc.) and education level?

**4. What are they working for?**

*Probes:* (1) Describe these jobs? (2) The reasons that they come here?

**5. Where is the Chinese migrants' living community in Nairobi, Kenya?**

*Probes:* (1) Where do they live? (2) Who do they live with? Are they usually living with their family members?

**6. How are their feelings about Nairobi, Kenya? Will they plan to permanently resident here?**

*Probes:* (1) If yes, why? (2) If not, why?

**7. How do Chinese migrants social network here? Is there any social network place or platform here?**

*Probes:* (1) If yes, give some examples. (2) If not, why?

**The Health Status of Chinese Migrants**

**8. Did Chinese migrants here have some health-related problems? (e.g. injury, infectious diseases, fevers, etc.)**

*Probes:* (1) What is the problem? (2) How do they deal with that?

**9. Did Chinese migrants here have some chronic diseases problems? (e.g. hypertension, diabetes, etc.)**

*Probes:* (1) What is the problem? (2) How do they deal with that?

**The Health Services Utilization of Chinese Migrants**

**10. Where do Chinese migrants get the medicine in Nairobi, Kenya?**

*Probes:* (1) Where to get the medicine? (2) Where to buy medicine here?

**11. How do Chinese migrants seek health services here?**

*Probes:* (1) What kind of health facilities do Chinese migrants prefer to go usually? (2) Why choose these facilities? (3) How's the quality of these health facilities? (4) Does it able to resolve their health problems? (5) How do Chinese migrants pay for the services there? How about the price?

**12. Do Chinese migrants have health insurance for health services in Kenya?**

*Probes:* (1) What kinds of health insurance? (2) Will these facilities accept that?

**13. Do you know any resources or supports from China or Kenya are able to help Chinese migrants’ health utilization in Kenya? (e.g. clinics, services, insurance, payment, etc.)**

*Probes:* (1) Health insurance or reimbursement? (2) health policy or measures?

**14. What is the biggest barrier or difficulty for health seeking or utilization for Chinese migrants in Kenya?**

*Probes:* (1) Barriers or difficulties for self-treatment? (2) Barriers or difficulties for health services seeking?

**15. What is the health utilization problem pressed for solution for Chinese migrants in Kenya?**

*Probes:* (1) For self-treatment (2) For health services seeking
